# New highlights in cancer and depression multimorbidity: a scoping systematic review

**DOI:** 10.3389/fonc.2025.1674653

**Published:** 2025-12-02

**Authors:** Lin Tang, Danyang Li, Minghua Wu

**Affiliations:** 1 Xiangya School of Public Health, Central South University, Changsha, Hunan, China; 2 The Affiliated Cancer Hospital of Xiangya School of Medicine, Central South University, Changsha, Hunan, China; 3 Hunan Cancer Hospital, Central South University, Changsha, Hunan, China; 4 The Key Laboratory of Carcinogenesis of the Chinese Ministry of Health, The Key Laboratory of Carcinogenesis and Cancer Invasion of the Chinese Ministry of Education, Cancer Research Institute, Central South University, Changsha, Hunan, China

**Keywords:** cancer, depression, risk factors, inflammation, multimorbidity

## Abstract

**Introduction:**

The co-occurrence of cancer and depression represents a prevalent and clinically significant form of multimorbidity, associated with poorer prognosis and increased healthcare burden. Despite this, current care models often operate in silos, resulting in fragmented management between oncology and psychiatry. This scoping review systematically maps existing evidence on cancer–depression multimorbidity to clarify epidemiological associations, elucidate underlying pathophysiological mechanisms, and synthesize integrated management strategies.

**Methods:**

The review followed the Joanna Briggs Institute methodology for scoping reviews and adhered to the PRISMA-ScR reporting guidelines. A systematic search was conducted across four electronic databases (PubMed, Scopus, Web of Science, and Embase) for studies published between 2020 and 2025. Eligible studies included adult populations with cancer–depression multimorbidity, addressing epidemiology, mechanisms, or management outcomes.

**Results:**

From 11,803 initial records, 36 studies met the inclusion criteria. The evidence consistently indicates a significant association between depression and increased risk of both all-cause and cancer-specific mortality across multiple cancer types. The included studies demonstrated notable heterogeneity in depression assessment methods and a geographical concentration in Asia, Europe, and North America.

**Discussion:**

This scoping review establishes a substantial and consistent body of evidence linking depression to elevated mortality risk in patients with cancer, identifying depression as a critical and modifiable prognostic factor. The synthesis highlights key evidence gaps, including the underrepresentation of low- and middle-income countries and variability in depression measurement. These findings emphasize the need for systematic integration of depression screening and management into routine oncologic care and call for future research to develop standardized assessment tools and culturally adapted intervention models.

## 1 Introduction

In 1970, Feinstein defined “comorbidity” as “any distinct additional entity that has existed or may occur during the clinical course of a patient who has the index disease under study” (1). Since 1976, the term “multimorbidity” has been increasingly adopted by health researchers to describe patients with multiple chronic conditions (2, 3). Today, multimorbidity generally refers to “the co-occurrence of multiple chronic or acute diseases and medical conditions within one person,” without reference to an index condition (4). A well-documented example is the coexistence of diabetes and cardiovascular disease. These conditions share common risk factors such as obesity and insulin resistance and involve overlapping pathological processes (5, 6). Their coexistence leads to higher mortality, reduced functional status and quality of life, and greater dependence on healthcare services (7).

Cancer frequently coexists with other medical conditions. Among these, depression stands out as the most common psychological multimorbidity, with a global pooled prevalence of 27% (95% confidence interval: 24–30%) (8, 9). The coexistence of cancer and depression poses a significant clinical challenge. Patients with cancer who experience depression have a 25% higher all-cause mortality risk than those without depression (10), and 29% of those with major depressive disorder report suicidal ideation (11). Therefore, managing this multimorbidity is crucial. Effective management not only improves patient survival but also enhances the efficiency of healthcare resource utilization.

In this context, conducting a scoping review is essential to systematically consolidate the existing but fragmented literature on this multimorbidity. This review aims to delineate the scope and strength of the epidemiological association, synthesize current knowledge on shared pathophysiological mechanisms, and critically evaluate the evidence supporting integrated management strategies. By mapping this landscape, the review will identify key knowledge gaps and guide future research and clinical practice.

## 2 Methods

The review was conducted following the established methodological framework by Arksey & O’Malley (2005) (12), incorporating the enhancements proposed by Levac et al. (2010) (13). The reporting process strictly adhered to the Preferred Reporting Items for Systematic reviews and Meta-Analyses extension for Scoping Reviews (PRISMA-ScR) guidelines and flow diagram (14).

### 2.1 Study design

This study employed a scoping review methodology, structured around the five-stage framework proposed by Arksey & O’Malley (2005). The stages include: (1) identifying the research question; (2) identifying relevant studies; (3) selecting studies for inclusion; (4) charting the data; and (5) collating, summarizing, and reporting the results.

### 2.2 Research question

This scoping review is guided by the following overarching question: What is the nature and extent of the existing evidence regarding the association between depression and mortality in cancer patients?

### 2.3 Eligibility criteria

To ensure focus and precision, clear inclusion and exclusion criteria were established, guided by the PCC (Population, Concept, Context) framework.

Inclusion criteria:

1. Population: Adult patients diagnosed with any type of cancer;
2. Concept: Studies must assess the association between depression and mortality or survival rates;
3. Context: The outcomes of interest are limited to all-cause mortality, cancer-specific mortality, or survival rates such as overall survival and disease-free survival;
4. Study Design: Observational studies;
5. Publication Date: To examine the latest research and identify recent advancements in the field, the study period is limited to January 2020 through October 2025;
6. Language: Articles providing an English abstract or full text.

Exclusion criteria:

1. Population: Non-cancer populations or animal studies;
2. Concept: Studies that do not link depression with the pre-defined death/survival outcomes;
3. Context: Publications outside the specified date range or without accessible English abstracts.

### 2.4 Search strategy

Prior to initiating the review, a preliminary search was conducted to confirm the absence of published or registered reviews on this specific topic. Following team discussions, four electronic databases were selected for the systematic search: PubMed, Scopus, Web of Science, and Embase. The search strategy combined controlled vocabularies and free-text keywords tailored to the syntax requirements of each database. Keywords included: “cancer”, “neoplasms”, “depression”, “depressive disorder”, “mortality”, “survival”, “prognosis”, etc. All searches were performed independently by two researchers and cross-checked. The full search strategy is available in the **Supplementary Materials**.

### 2.5 Study selection

The study selection process involved three stages: (1) Deduplication: Automated removal of duplicates using reference management software, supplemented by manual verification; (2) Title/Abstract Screening: Two researchers independently screened titles and abstracts based on the PCC criteria; (3) Full-Text Assessment: Potentially eligible articles underwent full-text review to confirm compliance with all inclusion criteria. Discrepancies at any stage were resolved through discussion or arbitration by a third researcher.

### 2.6 Charting the data and quality assessment

Data extraction was independently performed by two researchers using a pre-designed form. Disagreements were resolved through consensus or third-party arbitration. Extracted information included: basic study details (author, year, country, study design), sample characteristics (cancer type, sample size, demographics), depression measurement tool, survival/mortality outcome indicators, and effect estimate of their association with depression.

The methodological quality of the included non-randomized studies was independently assessed by two researchers using the Newcastle-Ottawa Scale (NOS) (15), which evaluates studies across three domains: selection, comparability, and outcome. Any disagreements were resolved through consultation with a third researcher. Studies with a score of ≥f on the 9-point scale were considered of sufficient quality for inclusion. The detailed results of this quality assessment are presented in **Supplementary Table S3** in **Supplementary Material**.

## 3 Results

### 3.1 Characteristics of included studies

**Figure 1:**
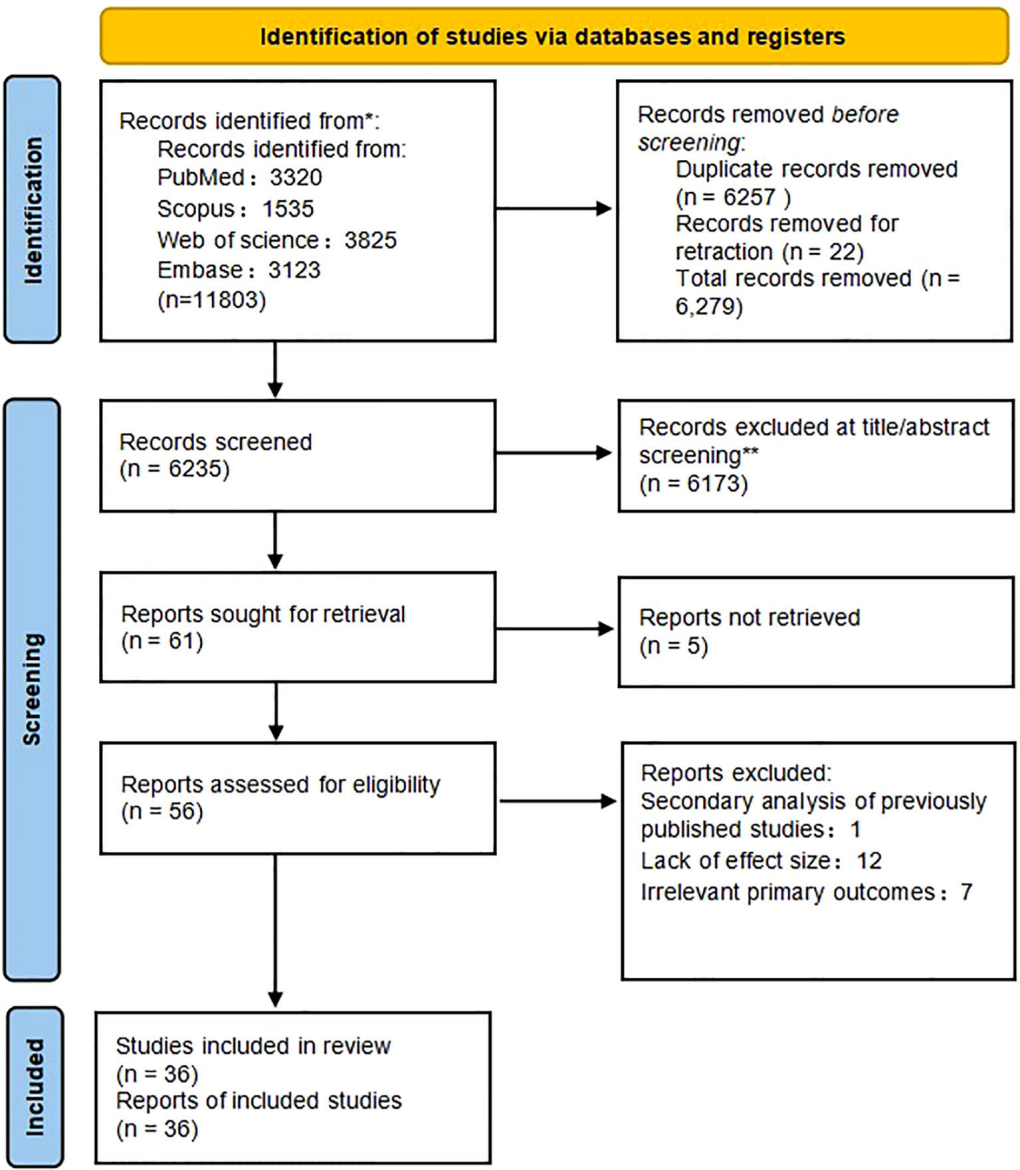
Flowchart of the search and selection process.

The systematic search of PubMed, Scopus, Web of Science, and Embase initially yielded 11,803 potentially relevant records. After removing duplicates and retracted publications, 6,235 records remained for title and abstract screening. Of these, 6,173 were excluded for not meeting the inclusion criteria. Full texts were retrieved and assessed for 62 studies, resulting in the final inclusion of 36 studies that provided complete data, appropriate designs, and relevant primary outcomes. The remaining studies were excluded for being secondary analyses, lacking effect estimates, or addressing unrelated outcomes. The detailed selection process and reasons for exclusion are summarized in **Figure 1**, and comprehensive information on each included study is provided in the **Supplementary Materials**.

The included studies originated from diverse geographical regions, primarily China (n = 8) (16–23)and the United States (n = 12) (24–35), many of which utilized multicenter or national databases. Additional contributions also came from the United Kingdom (n = 5) (36–40), South Korea (n = 3) (41–43), Spain (n = 3)(n = 3) (44–46), Italy (n = 2) (47, 48), Sweden (n = 2) (49, 50), and Canada (n = 1) (51).

Sample sizes varied substantially across studies, ranging from 104 to 258,259 participants, with an approximate mean of 29,809 per study (across 36 studies). Smaller studies typically focused on specific cancer types or single-center cohorts, whereas larger studies commonly drew on national registries or insurance databases. All included studies reported their sample sizes, with no missing data.

The study populations encompassed a wide range of cancer types, most frequently breast (n = 6), colorectal (n = 4), and prostate cancer (n = 3). Additional malignancies included ovarian, brain, bladder, lung, lymphoma, cervical, esophageal, gastric, pancreatic, and head and neck cancers. This distribution reflects the extensive investigation of psychological factors across diverse malignancies. Moreover, seven studies included multiple cancer types or community-based cohorts, further enhancing the generalizability of the findings.

The assessment of psychological factors varied across studies. Most investigations focused on depression and anxiety, defined through standardized rating scales or clinical diagnostic codes. Reported exposures included depressive symptoms (n = 28), anxiety symptoms (n = 22), psychological distress (n = 5), and social isolation (n = 1). Several studies additionally examined antidepressant use (n = 6) or inflammatory biomarkers (n = 2) in auxiliary analyses. Prognostic outcomes primarily included all-cause mortality (n = 25) and cancer-specific mortality (n = 12), with additional outcomes such as progression-free survival, overall survival, and recurrence rates.

The evidence base comprised only observational studies, with the vast majority adopting cohort designs (n = 34). Among these, 21 were retrospective—typically employing registries or databases—and 13 were prospective. The remaining studies included one retrospective case-control and one retrospective case-cohort design.

### 3.2 Increased mortality risk

Depression is a strong predictor of increased mortality risk, a finding consistently observed across various cancer types and large cohort studies (**Table 1**). In a large prospective cohort involving 20,582 patients with five common cancers (breast, colorectal, gynecological, lung, and prostate), the pooled hazard ratio for all-cause mortality among those meeting criteria for major depressive disorder was 1.41 (95% confidence interval: 1.29–1.54) (39). This association was independently confirmed across multiple malignancies, including both solid tumors and hematologic cancers. Even depressive symptoms identified through standardized scales represented a significant risk factor. In a study of Chinese patients with ovarian cancer, individuals with negative emotions (assessed using the Self-Rating Depression Scale and Self-Rating Anxiety Scale) had significantly lower two-year (65.1% vs. 80.9%) and three-year (44.2% vs. 65.1%) survival rates compared with those without such symptoms. Multivariate analysis further confirmed that negative emotion was an independent adverse prognostic factor (odds ratio = 0.256) (17). Moreover, the coexistence of depression with other psychosocial stressors further magnified mortality risk. Among patients with breast cancer in the United Kingdom, those with both depression and sleep disorders had a 75% higher risk of death compared with those with neither condition (36).

**Table 1:** Evidence from included studies on depression and increased mortality risk in cancer patients.

Authors	Year	Country	Study design	Study population	Exposure factors	Outcome measures	Key findings/effect measures
Yang G et al. (16)	2022	China	Retrospective cohort study	234 Pituitary adenoma (PA) patients undergoing surgery	SDS > 52 defined as depression	Cure/effective/recurrence	Perioperative depressive symptoms (SDS >52) was an independent risk factor for poor prognosis in pituitary adenoma patients (Adjusted OR = 2.504, 95% CI: 1.418–7.458, *p* = 0.003).
He J, Zhang Y (17)	2023	China	Retrospective cohort study	258 ovarian cancer patients undergoing surgery	SDS > 52 or SAS > 50 defined as negative emotion	2-year/3-year survival rate, recurrence rate	Perioperative negative emotions were independently associated with poorer survival, with lower 2-year (65.1% vs 80.9%) and 3-year (44.2% vs 65.1%) survival rates (Adjusted OR = 0.256, 95% CI: 0.098–0.672, *p* = 0.006).
Trudel-Fitzgerald C et al. (34)	2020	USA	Prospective cohort study	1,732 colorectal cancer patients	Comprehensive anxiety/depression symptoms, diagnosis, medication use	All-cause mortality, CRC-specific mortality	Both anxiety and depression symptoms were associated with increased all-cause mortality (HR per 1-SD increase: 1.16 for each). Clinical depression was significantly linked to higher mortality (HR = 1.28, 95% CI: 1.06–1.56).
Shim EJ et al. (41)	2020	South Korea	Retrospective cohort study	124,381 breast cancer patients	ICD-10 diagnosis of depression/anxiety, antidepressant treatment	All-cause mortality	Depression (HR = 1.26, 95%CI 1.18-1.36), anxiety (HR = 1.14, 95% CI 1.08-1.22), and their comorbidity (HR = 1.38, 95% CI 1.24-1.54) were associated with increased all-cause mortality. Antidepressant treatment was associated with a reduction in this excess risk.
Paredes AZ et al. (31)	2021	USA	Population-based retrospective cohort study	54,234 pancreatic cancer patients	ICD diagnosis of mental illness (depression, anxiety, etc.)	All-cause mortality, pancreatic cancer-specific mortality	Patients with pre-existing mental illness, particularly severe disorders (bipolar/schizophrenia), had lower rates of curative surgery and higher all-cause (HR = 1.20, 95% CI 1.21-1.40) and pancreatic cancer-specific (HR = 1.27, 95% CI 1.17-1.37) mortality.
Rumalla K et al. (32)	2020	USA	National retrospective cohort study	57,621 malignant brain tumor surgeries	ICD-9 diagnosis of major depressive disorder	Complications, readmission rates	The presence of MDD was associated with nonroutine discharge (odds ratio, 1.10-125; *p* < 0.0001) as well as higher rates of neurologic complications (odds ratio, 1.03-1.18; *p* = 0.003).
Tao F et al. (18)	2023	China	Prospective cohort study	178 advanced gastric cancer patients receiving chemotherapy	SAS/SDS > 50 defined as negative emotions	PFS, OS, quality of life	Negative emotions were prevalent and associated with shorter PFS and OS. They were an independent risk factor for OS (HR = 0.702, 95%CI=0.497- 0.997, *p* = 0.045) and were linked to worse quality of life.
Ouh YT et al. (43)	2025	South Korea	National retrospective cohort study	85,327 gynecologic cancer patients	ICD-10 diagnosis of depression/anxiety	All-cause mortality	Depression alone (OR = 1.46, 95% CI 1.27-1.66) and comorbid depression/anxiety (OR = 1.47, 95% CI 1.31-1.65) were independent predictors of higher all-cause mortality. Anxiety alone was not significantly associated with mortality.
Tan PX et al. (20)	2025	China	Single-center retrospective cohort study	319 esophageal cancer MIE patients	PHQ-9 assessed postoperative depression	Recurrence-free survival	LASSO and Cox regression identified clinical stage (HR = 2.472, *p* = 0.003), the preoperative systemic inflammatory index (SII, HR = 1.001, P<0.001), and depressive symptoms severity (HR = 2.398, *p* = 0.004) as independent predictors of RFS.
Sanghvi DE et al. (35)	2024	USA	Prospective cohort study	2,342 cancer patients	CES-D assessed depression trajectories (four types)	Mortality	Longitudinal depressive symptoms trajectories were strong predictors of mortality. The incident (worsening) trajectory carried the highest risk (OR = 6.89, 95% CI = 4.55-10.43), followed by recovering and chronic trajectories.
Sancassiani Fet al. (48)	2021	Italy	Longitudinal cohort study	263 cancer patients	PHQ-9 defined as MDD	Early death within 9 months	MDD was associated with a significantly increased risk of premature death within 9 months (RR = 2.15, 95% CI: 1.10–4.20) in a mixed cancer cohort.
Herweijer E et al. (50)	2023	Sweden	National registry-based cohort study	20,177 cervical cancer patients	ICD diagnosis of mental disorders	All-cause mortality, cervical cancer-specific mortality	Pre-existing mental disorders were associated with worse overall survival (fully adjusted HR = 1.19, 95% CI, 1.06-1.34). The association with cervical cancer-specific mortality was attenuated after full adjustment for cancer stage and sociodemographics.
Leung B et al. (51)	2021	Canada	Retrospective cohort study	25,382 geriatric cancer patients	PSSCAN-R assessed anxiety/depression/social isolation	Overall survival	Anxiety (HR = 1.30, 95%CI=1.24–1.37), depressive symptoms (HR = 1.51, 95%CI=1.43–1.59), and social isolation (HR = 1.12, 95%CI=1.07–1.67) were independent predictors of shorter overall survival in geriatric cancer patients.
McFarland DC et al. (30)	2021	USA	Retrospective case cohort study	123 metastatic lung cancer patients	HADS-D defined as depression, CRP mg/dl as inflammation	Overall survival	Both depressive symptoms (HR = 1.12, 95% CI:1.05-1.179) and inflammation (CRPamm HR = 2.85, 95% CI:1.856-4.388) were independent risk factors for shorter survival. Depression partially mediated the effect of inflammation on survival.
Gallagher TJ et al. (27)	2025	USA	Retrospective cohort study	258,259 HNC survivors	ICD diagnosis of anxiety/depression, treatment modalities	Mortality	Treatment for anxiety/depressive symptoms, particularly psychotherapy (HR = 0.75, 95% CI:0.68-0.82), was associated with a significant reduction in all-cause mortality among HNC survivors.
Kuczmarski TM et al. (28)	2023	USA	Population-based retrospective cohort study	13,244 DLBCL patients	ICD-9-CM diagnosis of depression/anxiety	5-year overall survival, lymphoma-specific survival	Pre-existing depression was a significant independent predictor of markedly inferior survival in patients with DLBCL. Those with depression alone faced the greatest risk, with a 37% increase in all-cause mortality (HR 1.37, 95% CI 1.28-1.47) and a similarly elevated 37% increase in lymphoma-specific mortality (HR 1.37, 95% CI 1.26-1.49) compared to patients with no mental health disorder.

CI, Confidence Interval; RR, Relative Risk; ICD, International Classification of Diseases; SAS, Self-Rating Anxiety Scale; DIS, Diagnostic Interview Schedule; PHQ-9, Patient Health Questionnaire-9; HADS, Hospital Anxiety and Depression Scale; SDS, Self-Rating Depression Scale; BDI-II, Beck Depression Inventory-II; DLBCL, diffuse large B-cell lymphoma; HNC, Head and Neck Cancer; PSSCAN-R, the Revised Psychosocial Screen for Cancer (PSSCAN-R) questionnaire.

The severity and longitudinal course of psychological distress provide stronger prognostic value than a single baseline assessment. Several studies demonstrated a clear dose–response relationship; for example, among patients with colorectal cancer, each one-point increase in the depression scale score was associated with an 11.8% higher risk of death within the following year (44). More importantly, dynamic monitoring of depressive symptom trajectories enables the identification of patients at particularly high risk. In a prospective study conducted in the United States, patients categorized in the “emergent” trajectory group—those with progressively worsening depressive symptoms—had nearly a sixfold higher risk of death compared with the “resilient” group, characterized by consistently low symptom levels (35).

Potential mediating mechanisms involve both behavioral and biological pathways, with reduced treatment adherence and systemic inflammation representing two principal routes. On the behavioral level, a nationwide U.S. database study on bladder cancer demonstrates that patients with mental illness—primarily depression—were significantly less likely to receive guideline-recommended definitive treatment, directly contributing to poorer survival outcomes (33). On the biological level, research has revealed intrinsic links between depression and physiological processes. In a study of patients with metastatic lung cancer, depression partially mediated the relationship between systemic inflammation, measured by C-reactive protein levels, and reduced survival (30). Importantly, evidence indicates that addressing psychological disorders can mitigate mortality risk, offering a clear avenue for clinical intervention. A large U.S. retrospective study of head and neck cancer survivors found that pharmacologic and/or psychotherapeutic treatment for anxiety or depression was associated with a significant reduction in mortality risk, with psychotherapy alone demonstrating the strongest protective effect (27). These findings suggest that psychological factors are not merely prognostic indicators but also modifiable therapeutic targets.

## 4 Discussion

The evidence synthesized in this scoping review consistently demonstrates a strong association between depression, in its various manifestations, and an increased risk of mortality among patients with cancer. This relationship persists across diverse cancer types and applies to both all-cause and cancer-specific mortality. Although these quantitative findings are compelling, they raise several critical and interrelated questions concerning the underlying mechanisms, moderating influences, and clinical implications of this association. The following discussion builds on these core results to examine the biological and behavioral pathways that may explain the observed link, situate the findings within the broader body of evidence, and outline the key clinical and research priorities that emerge from this work.

### 4.1 Pathophysiology of multimorbidity

**Figure 2:**
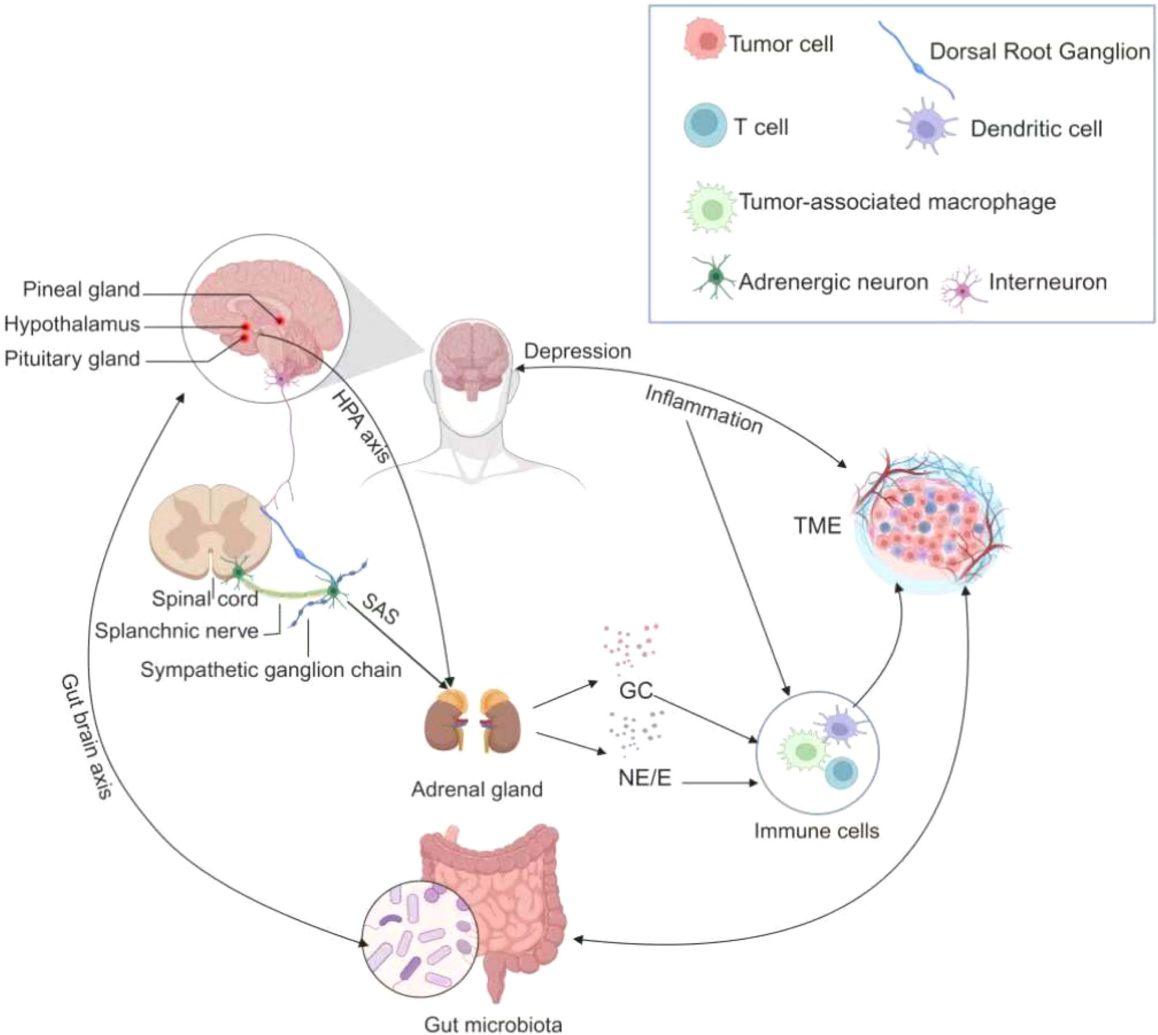
The pathophysiological mechanisms in multimorbidity of cancer and depression. The figure illustrates the intricate pathophysiologic relationships in individuals with co-morbid cancer and depression. Tumor cells release inflammatory cytokines that traverse the blood-brain barrier, thereby precipitating depressive symptoms. Subsequently, depression stimulates the Hypothalamic-pituitary-adrenal axis (HPA axis) and the Sympathetic-adrenal-medullary system (SNS), prompting the adrenal glands to secrete glucocorticoids and catecholamines. These stress hormones can influence tumor progression by interacting with various cell types in the tumor microenvironment. Furthermore, a bidirectional relationship exists between the gut microbiome and both cancer and depression. These multifaceted mechanisms collectively establish a pathophysiologic link between the co-morbidities of cancer and depression.

The multimorbidity of cancer and depression arises from complex interactions among inflammatory, neuroendocrine, immune, and neurotransmitter systems (**Figure 2**). These systems are interconnected through overlapping biological pathways that collectively create a synergistic network driving both the onset and progression of this comorbid state.

#### 4.1.1 Inflammation

##### 4.1.1.1 Inflammatory cytokines

Toll-like receptors recognize pathogen-associated and damage-associated molecular patterns released from necrotic or tumor cells. Activation of these pattern recognition receptors triggers intracellular signaling cascades through adaptor proteins, leading to the activation of transcription factors such as nuclear factor kappa-light-chain-enhancer of activated B cells (NF-κB) and signal transducer and activator of transcription 3 (STAT3) (52). These pathways regulate the production of inflammatory cytokines and type I interferons. Under physiological conditions, inflammatory cytokines support neurogenesis; however, in malignancy, their overexpression induces oxidative stress, excessive glutamate release, impaired neurogenesis, and disrupted neuroplasticity (53), thereby contributing to the development of depression (54). In addition, activation of the interleukin (IL)-6/STAT3 pathway promotes tumor survival through the upregulation of anti-apoptotic myeloid cell leukemia sequence 1 (55, 56) and cyclin A1 (57), while also stimulating downstream expression of S100 calcium-binding protein A9 (S100A9). S100A9, a key mediator of tumor invasion and metastasis, enhances tumor cell migration and invasiveness (58).

##### 4.1.1.2 Tryptophan metabolism pathway

**Figure 3:**
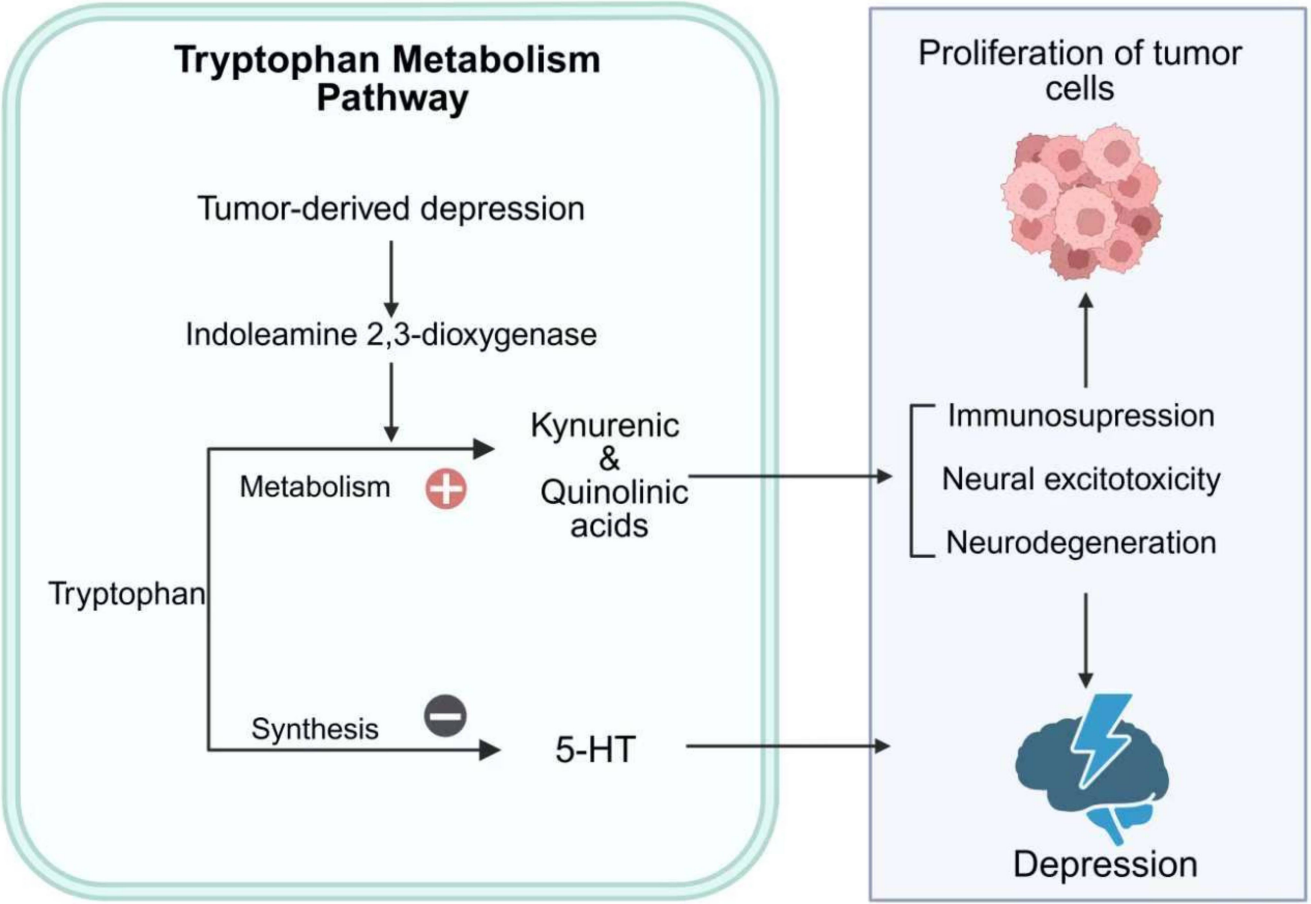
Tryptophan metabolism in cancer-depression multimorbidity. This figure illustrates the role of tryptophan metabolism in the multimorbidity of cancer and depression. Tumor-derived depression activates IDO, shifting tryptophan metabolism toward the production of kynurenic and quinolinic acids. This metabolic shift reduces the synthesis of 5-HT, contributing to depressive symptoms. Additionally, the accumulation of kynurenic and quinolinic acids promotes immunosuppression, neural excitotoxicity, and neurodegeneration, which can exacerbate both tumor cell proliferation and depression. The figure highlights the bidirectional interaction between cancer and depression through tryptophan metabolic pathways.

Tryptophan metabolism plays a pivotal role in the multimorbidity of cancer and depression (**Figure 3**). Inflammatory cytokines can upregulate the expression of indoleamine 2,3-dioxygenase through activation of STAT1α, NF-κB, and p38 mitogen-activated protein kinase (MAPK) signaling pathways (59). Indoleamine 2,3-dioxygenase catalyzes the degradation of tryptophan, a precursor of serotonin, into kynurenine metabolites such as kynurenic acid and quinolinic acid, thereby disrupting 5-hydroxytryptamine neurotransmission (60). These metabolites exert neurotoxic effects, promoting excitotoxicity and neurodegeneration that contribute to depressive symptoms (61). Beyond neurotransmitter dysregulation, indoleamine 2,3-dioxygenase also modulates the tumor microenvironment by suppressing CD8+ tumor-infiltrating lymphocytes and promoting cancer cell survival, proliferation and invasion (61).

##### 4.1.1.3 Neurotransmitters and neurotrophic factors

Inflammatory cytokines are believed to alter synaptic monoamine neurotransmission through multiple mechanisms, contributing to the pathophysiology of depression. IL-1β and tumor necrosis factor activate the p38 MAPK pathway, leading to increased expression and function of serotonin reuptake transporters (76). Inflammatory cytokines also reduce the expression of glial glutamate reuptake transporters while stimulating glutamate release from glial cells (77). The resulting elevation in extracellular glutamate promotes excessive activation of extrasynaptic N-methyl-D-aspartate receptors, causing excitotoxicity and reduced production of brain-derived neurotrophic factor (78). As brain-derived neurotrophic factor is essential for neurogenesis and antidepressant response, its downregulation links inflammation to mood disturbances. In stress-induced depression animal models, reductions in inflammatory cytokines and downstream signaling activity have been observed, highlighting the dynamic interaction between inflammation and neurotransmission (79, 80).

#### 4.1.2 Chronic stress

##### 4.1.2.1 Hypothalamic–pituitary–adrenal axis dysregulation

The continuous production of inflammatory cytokines such as IL-6 and tumor necrosis factor by tumors leads to persistent activation of the hypothalamic–pituitary–adrenal axis (62). Chronic stimulation of this axis contributes to glucocorticoid resistance and impaired negative feedback regulation, thereby perpetuating systemic inflammation (63). Dysregulation of the hypothalamic–pituitary–adrenal axis can facilitate tumor progression through the upregulation of signaling molecules such as fibroblast growth factor 2 and activation of epithelial–mesenchymal transition pathway (64). Epithelial–mesenchymal transition, a tightly regulated developmental program, drives the invasion–metastasis cascade (65). Through this process, epithelial cells acquire enhanced motility, resistance to apoptosis, and the capacity for dissemination and colonization at distant sites (66).

##### 4.1.2.2 Sympathetic overdrive

Chronic activation of the sympathetic nervous system promotes tumor progression through β-adrenergic signaling. Norepinephrine and epinephrine bind to β-adrenergic receptors, activating downstream pathways such as p38 MAPK, which contributes to abnormal cancer cell proliferation (67, 68). These catecholamines also induce DNA damage and suppress p53 expression via Gs–protein kinase A and β-arrestin-mediated signaling, leading to the accumulation of genetic damage (69). As p53 is a critical tumor suppressor protein, its loss impairs cell cycle arrest and DNA repair mechanisms, promoting uncontrolled tumor cell proliferation (70). Beyond direct effects on cancer cells, catecholamines modulate the tumor microenvironment by inducing vascular endothelial growth factor (VEGF) expression. Norepinephrine stimulates VEGF production through β-adrenergic receptor activation on vascular endothelial cells (71, 72). VEGF subsequently binds to its receptors, activating downstream signaling cascades such as the phosphatidylinositol 3-kinase–protein kinase B and Rat sarcoma–Rapidly accelerated fibrosarcoma–mitogen-activated protein kinase kinase–extracellular signal-regulated kinase pathways, which promote endothelial cell proliferation, migration, and survival—key steps in tumor angiogenesis (73–75). Furthermore, VEGF increases vascular permeability, facilitating tumor cell intravasation and distant metastasis (76).

#### 4.1.3 Immune evasion

T cells are central mediators of the immune response, providing potent defense against infections and malignancies. They coordinate cell-mediated immunity through antigen-specific recognition, direct cytotoxicity, and cytokine release, which amplifies broader immune responses (77). T cell activity is tightly regulated by inhibitory checkpoints such as cytotoxic T-lymphocyte-associated antigen 4, programmed cell death protein 1, and B- and T-lymphocyte attenuator, which prevent uncontrolled proliferation, collateral cytotoxic damage, and autoimmunity (78). Tumor cells exploit these regulatory pathways by expressing immune checkpoint molecules and their corresponding ligands—B7 for cytotoxic T-lymphocyte-associated antigen 4 and programmed death-ligand 1 for programmed cell death protein 1—on their surface or on antigen-presenting cells. These ligand–receptor interactions suppress T cell activation, allowing tumors to evade immune surveillance. Blockade of immune checkpoints restores T cell function and facilitates tumor eradication, forming the basis of modern immunotherapies (79).

**Figure 4:**
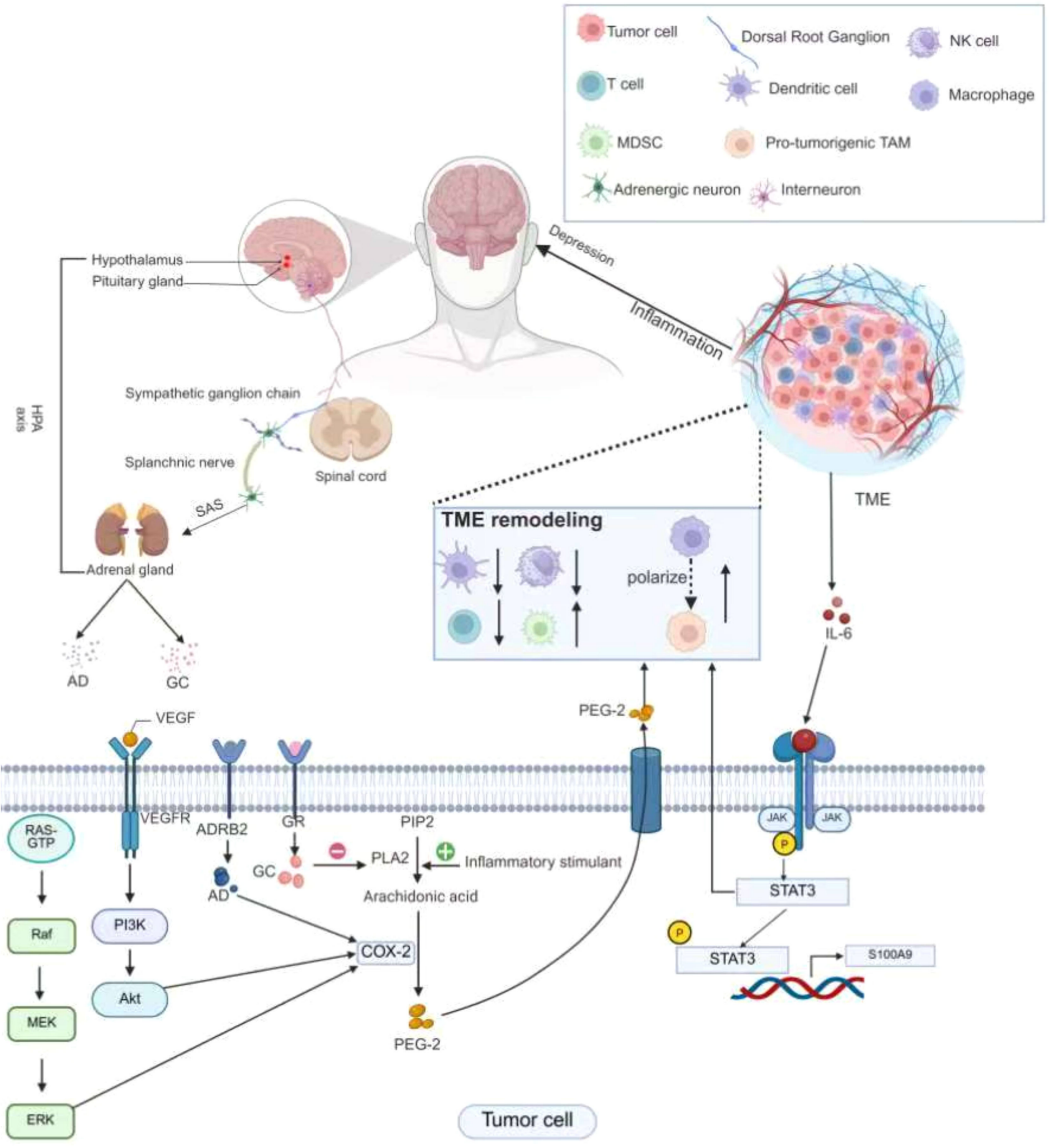
Mechanisms of tumor immune evasion. This figure outlines how depression induces tumor cell immune evasion via the HPA axis (cortisol), SNS (epinephrine), and pro - inflammatory cytokines. Corticosteroids and upregulated glucocorticoid - induced factor Tsc22d3 block the type I IFN response in DCs and IFN-o + T cells. IL - 6 inhibits DC function and T - cell immune responses, and promotes immunosuppressive cell accumulation via STAT3. Epinephrine induces COX - 2, activating the COX - 2/PGE2 pathway, which remodels the lymphatic network around tumors and inhibits T - cell function.

However, emerging evidence indicates that depression is associated with poorer outcomes among patients with cancer receiving immunotherapy (80). This may reflect depression-mediated mechanisms of tumor immune evasion (**Figure 4**). First, elevated plasma corticosteroid levels and upregulation of the glucocorticoid-induced leucine zipper protein TSC22 domain family protein 3 suppress the type I interferon response in dendritic cells and interferon gamma-producing T cells, thereby impairing immunosurveillance and reducing the efficacy of antitumor therapies in non-small-cell lung cancer and colorectal cancer (81). Second, epinephrine induces cyclooxygenase-2 (COX-2) and COX-2-dependent inhibitors in tumor cells, activating the COX-2/prostaglandin E2 signaling pathway (82). Activation of the rapidly accelerated fibrosarcoma–mitogen-activated protein kinase kinase and phosphatidylinositol 3-kinase signaling pathways further upregulates COX-2 expression in tumor cells (83, 84). The COX-2/prostaglandin E2 axis remodels the peritumoral lymphatic network, suppresses T cell activity, and enhances tumor immune evasion (85, 86). Moreover, depression promotes tumor-associated macrophage infiltration through neuropeptide Y signaling and accelerates tumor progression via activation of the IL-6/STAT3 pathway (87). Activation of IL-6/STAT3 impairs dendritic cell antigen presentation, reduces T cell responses, and promotes the accumulation of immunosuppressive cell populations such as regulatory T cells and myeloid-derived suppressor cells, fostering an immunosuppressive tumor microenvironment (88, 89). Furthermore, IL-6-mediated STAT3 activation induces the expression of S100A9, a factor that promotes tumor invasion and metastasis (58). Collectively, these mechanisms illustrate how depression can contribute to tumor immune evasion, undermining antitumor immunity and compromising therapeutic efficacy.

#### 4.1.4 Gut microbiota

The gut microbiota, a complex community of microorganisms inhabiting the gastrointestinal tract, represents an important environmental factor influencing human physiology and disease susceptibility (90). Growing evidence suggests that alterations in gut microbial composition can act as either risk or protective factors in the multiple diseases, including cancer. The gut microbiome has been particularly implicated in the pathogenesis of colorectal cancer, where dysbiosis contributes to tumor initiation and progression (91). Preclinical studies indicate that the gut microbiota may promote carcinogenesis and tumor progression through several mechanisms: (1) production of bacterial toxins that directly induce DNA damage; (2) generation of harmful metabolites derived from a Western-style diet; (3) chronic inflammation caused by microbial interactions with intestinal epithelial cells; (4) persistent infection or invasive biofilm formation; and (5) suppression of antitumor immune responses (92).

The gut microbiota also plays a crucial role in the bidirectional communication and shared pathophysiological mechanisms linking cancer and depression. Both tumorigenesis and depressive disorders are associated with gut microbial dysbiosis, which may exacerbate disease progression through interconnected immunological, metabolic, and neuroendocrine pathways (93). Cancer-related elevations in cortisol can disrupt intestinal barrier integrity, leading to increased permeability and chronic inflammation—hallmarks of the gut–brain axis dysfunction (94). Enhanced intestinal permeability (“leaky gut”) facilitates microbial translocation and the release of bacterial metabolites into systemic circulation, amplifying systemic inflammation. This process drives an imbalance between T helper 17 and regulatory T cells and elevates circulating levels of inflammatory cytokines such as IL-6, IL-1, and tumor necrosis factor, all of which are implicated in the pathogenesis of depression (94).

### 4.2 Behavioral determinants

#### 4.2.1 Therapeutic non-adherence and delayed care-seeking

Depression negatively influences treatment adherence through disruptions in cognitive control, motivation, and decision-making processes. Within the framework of the Theory of Planned Behavior, reduced adherence among patients with depression can be explained by diminished behavioral intention, negative expectations about treatment efficacy, and distorted perceptions of social norms (95, 96). At a neurobiological level, alterations in neuroplasticity—particularly aberrant functional connectivity between the prefrontal cortex and striatum—have been implicated in impaired decision-making and maladaptive health behaviors (97).

Empirical evidence supports the substantial impact of depression on adherence. Individuals with depression are approximately three times more likely to be non-adherent to medical recommendations compared with those without depression (98). Among cancer populations, patients with pre-existing depression or anxiety exhibit a significantly lower likelihood of receiving chemotherapy (odds ratio = 0.58, *p* = 0.04) (26). Depression also contributes to delayed care-seeking, further undermining adherence and reducing quality of life (99). In patients with advanced non-small-cell lung cancer, depression has been independently associated with poor treatment adherence and unfavorable prognosis (100). Non-adherence to therapeutic regimens and delays in seeking medical care may cause patients to miss critical treatment windows or limit their engagement in care, ultimately reducing therapeutic efficacy and worsening clinical outcomes.

#### 4.2.2 Cancer treatment-related toxicity

Conventional cancer therapies—including surgery, chemotherapy, and radiotherapy—remain central to the management of malignant tumors (101). Surgery directly removes tumor tissue, chemotherapy uses cytotoxic drugs to target rapidly dividing cancer cells, and radiotherapy employs ionizing radiation to damage DNA within malignant cells. Although these modalities are essential components of comprehensive cancer care, their associated toxicities can profoundly affect the physical and psychological well-being of patients (102). Treatment-related neurotoxicity is one of the most common adverse effects. Chemotherapy-induced peripheral neuropathy often causes chronic neuropathic pain (103), which heightens the risk of depression, anxiety, and sleep disturbances among patients with cancer (102). Radiotherapy also carries substantial psychosocial consequences. Between 25% and 50% of patients undergoing radiotherapy experience severe psychological distress and anxiety, and treatment-related toxicity may further exacerbate depressive symptoms (104, 105).

In recent years, newer modalities such as targeted therapy, gene therapy, and immunotherapy have been increasingly adopted in clinical oncology (106). Although these treatments offer improved efficacy and specificity, they are also associated with unique toxicities. Immunotherapy, for example, can trigger immune-related adverse events affecting the skin, endocrine glands, lungs, and heart (107). Despite their growing clinical use, the psychological impact of these toxicities remains underexplored and warrants systematic investigation.

#### 4.2.3 Stigma

Stigma, defined as the profound sense of shame or social discredit associated with a particular disease (108), is highly prevalent among patients with cancer (109). Its effects extend beyond emotional distress to directly influence health-seeking behavior. Stigmatization can discourage patients from seeking timely medical attention, leading to delays in diagnosis and treatment and ultimately compromising quality of life (110). Experiencing stigma is associated with numerous stressors, including health-related, occupational, familial, and financial challenges. A strong positive correlation has been documented between perceived stigma and the severity of depressive symptoms (111). Indeed, patients with cancer reporting higher levels of stigma also tend to exhibit more severe anxiety and depression (112). Visible treatment-related physical changes—such as alopecia, mastectomy, colostomy, or surgical scarring—often exacerbate social withdrawal and feelings of exclusion (113).

Given these effects, healthcare teams must approach cancer-related stigma with sensitivity and awareness. Delivering holistic cancer care requires recognizing that sociodemographic factors can influence the capacity of patients to cope with illness. Integrating stigma assessment and mitigation strategies into clinical practice can improve patient engagement, enhance treatment adherence, and elevate overall quality of care and satisfaction.

### 4.3 Shared risk factors and preventive strategies

The recognition that cancer and depression share overlapping biological mechanisms provides a strong rationale for re-examining their relationship through the lens of modifiable risk. This mechanistic convergence highlights the importance of developing actionable public health interventions targeting shared determinants across lifestyle, environmental, and socioeconomic domains that contribute to this multimorbidity.

#### 4.3.1 Diet

Nutrition plays a pivotal role in the etiology of both cancer and depression, representing a key shared risk factor for their co-occurrence. Unhealthy dietary habits—such as frequent consumption of high-fat foods, fried items, and processed meats—have been linked to an elevated risk of both oncogenesis and depressive disorders (114, 115). In colorectal cancer specifically, consumption of processed and red meats shows a significant positive association with cancer risk (116). Similarly, irregular eating behaviors, such as skipping or delaying breakfast, have been associated with a higher likelihood of developing affective disorders, whereas consistent, balanced eating patterns are linked to a markedly reduced risk (117).

Conversely, dietary patterns rich in plant-based foods and characterized by the Mediterranean diet have demonstrated protective effects against both cancer and depression. Increased intake of fruits and vegetables has been correlated with lower rates of depressive disorders (118, 119) and reduced incidence of several cancers, including pancreatic and bladder cancer (120, 121). Omega-3 polyunsaturated fatty acids have shown anti-inflammatory and neuroprotective properties, modulating neurotransmitter systems and cellular signaling cascades that may reduce susceptibility to both oncogenesis and mood disorders (122–125). Evidence from a large cohort study employing a dose–response model revealed a nonlinear relationship between fruit and vegetable consumption and breast cancer risk: disease risk decreased with intake up to approximately 90 servings per month but increased beyond this threshold, potentially owing to confounding factors such as total caloric intake (126). In summary, a diet rich in omega-3 polyunsaturated fatty acids, fruits, and vegetables may mitigate the shared biological and behavioral risks underlying cancer–depression multimorbidity. Such dietary interventions should be incorporated into a comprehensive preventive framework that includes regular physical activity, stress management, and routine medical evaluation. Future research should aim to clarify the specific mechanisms through which nutritional factors influence both oncogenesis and depression and determine how these insights can be translated into optimized preventive care protocols.

#### 4.3.2 Exercise

Physical inactivity is a major modifiable risk factor for both cancer and depression. Evidence indicates that sedentary behavior, particularly when it replaces light or moderate activity during adolescence, is associated with an increased risk of depressive symptoms (127). Furthermore, studies using genetic instruments have provided robust evidence for a protective association between objectively measured physical activity—but not self-reported activity—and the risk of major depressive disorder (128). This discrepancy likely arises because self-reported activity is prone to mood-congruent and recall biases, which can confound associations with mental health outcomes (128). Similarly, sedentary behavior has been independently linked to a higher risk of several cancers, including colon, endometrial, and lung cancers (129).

Exercise can be a cornerstone of preventive strategies for cancer–depression multimorbidity. Regular physical activity exerts beneficial effects on both physiological and psychological pathways implicated in these conditions. For instance, higher physical activity levels, as measured by the Physical Activity Scale for the Older Adult questionnaire, are associated with a lower risk of prostate cancer (130). Mechanistically, physical activity reduces systemic inflammation, improves insulin sensitivity, and optimizes body composition—all factors that decrease the risk of both oncogenesis and depressive disorders (131). Beyond its preventive role, physical activity is widely recognized for promoting overall health, preventing chronic diseases, and enhancing quality of life (132). Consistent engagement in exercise is associated with reduced incidence and improved outcomes across multiple cancer types (133). To mitigate the risk of cancer–depression multimorbidity, individuals should engage in regular, tailored exercise programs suited to their physical fitness and medical status. Practical and accessible modalities such as walking, cycling, and aquatic exercise offer effective means to maintain activity and support both physical and mental well-being.

#### 4.3.3 Weight management

Globally, obesity accounts for a substantial proportion of the cancer burden, with population-attributable fractions estimated at 11.9% in men and 13.1% in women (134). Elevated adiposity and the metabolic activity of excess adipose tissue have been strongly linked to the development of several cancers, including colorectal, pancreatic, renal, endometrial, postmenopausal breast, and esophageal adenocarcinoma (135, 136). Beyond its somatic consequences, obesity is closely associated with both psychological and metabolic disturbances. Individuals with obesity have an increased risk of depression, which may, in turn, exacerbate obesity through mechanisms such as appetite dysregulation, fatigue, and decreased physical activity (137).

Weight management influences multiple physiological and psychological pathways that underlie cancer–depression multimorbidity. Chronic inflammation and insulin resistance, characteristic of obesity, contribute to both tumor progression and the onset of depressive symptoms (138). Reducing these risk factors through weight loss can attenuate the biological processes linking obesity to both conditions. Moreover, weight management interventions typically include exercise and dietary modifications—both of which independently exert antidepressant and anticancer effects (139). Integrating weight management into comprehensive cancer prevention and survivorship strategies is therefore essential. Personalized lifestyle interventions that combine dietary modifications with structured physical activity can be tailored to the clinical and metabolic profile of each patient. For instance, weight loss programs incorporating calorie restriction and aerobic exercise have been shown to improve metabolic parameters and alleviate depressive symptoms in cancer survivors (140).

#### 4.3.4 Alcohol

Alcohol consumption represents a major shared behavioral risk factor connecting cancer and depression, acting through distinct yet interrelated biological and psychobehavioral mechanisms. From an oncologic perspective, alcohol is a well-established, modifiable carcinogen. In 2020, alcohol use accounted for an estimated 4.1% (approximately 741,000 cases) of all newly diagnosed cancer cases worldwide, with men comprising nearly three-quarters of these cases. The cancers most strongly associated with alcohol consumption include esophageal, liver, and female breast cancers (141, 142). The carcinogenic effects of alcohol are largely mediated through its metabolism to acetaldehyde, a toxic intermediate that promotes tumorigenesis via multiple interconnected pathways, including direct DNA damage, oxidative stress, lipid peroxidation, and chronic inflammation (143). In the context of mental health, numerous observational studies have reported a J- or U-shaped association between alcohol intake and depression risk, with both abstinence and heavy consumption linked to increased depressive symptomatology (144). The detrimental effects of heavy drinking, however, are unequivocal—it can precipitate or exacerbate psychiatric disorders and interfere with treatment adherence and recovery (145).

Given this well-established risk profile, primary prevention through the reduction or cessation of alcohol use is a critical public health priority. Despite clear evidence of harm, alcohol consumption remains frequently underrecognized and insufficiently addressed in oncologic care (146). Encouragingly, targeted interventions are effective: in a cohort of head and neck cancer survivors, patients who received explicit oncologist recommendations achieved significantly greater reductions in alcohol consumption (147). Therefore, systematic integration of alcohol screening, counseling, and brief interventions into routine oncology practice represents a practical and impactful strategy for mitigating both cancer progression and depression risk, ultimately improving overall patient outcomes.

### 4.4 Management of multimorbidity

The management of depression within the context of cancer–depression multimorbidity remains inadequate. Most existing clinical practice guidelines and healthcare delivery models are designed around single-disease management rather than integrated, patient-centered approaches (148). However, the timely identification and treatment of depression are essential to improving both psychological well-being and clinical outcomes in patients with cancer. Early integration of psychological interventions into oncologic care has been shown to reduce distress, improve treatment adherence, and facilitate the adoption of health-promoting behaviors (149, 150).

#### 4.4.1 Prudent use of antidepressant

The evidence linking antidepressant use to cancer outcomes remains complex, context-dependent, and likely influenced by both methodological and biological factors. Regarding cancer incidence, a meta-analysis reported a marginally increased risk of lung cancer among antidepressant users (151). However, this association was based on a limited number of studies, exhibited considerable heterogeneity (*I^2^* = 65.03%), and may have been confounded by unmeasured factors such as smoking. The relationship between antidepressant use and cancer-specific mortality presents an even more nuanced picture. Selective serotonin reuptake inhibitor use has been associated with higher mortality in breast cancer (152), whereas the use of any antidepressant has been linked to significantly reduced all-cause and cancer-specific mortality in patients with liver cancer (adjusted hazard ratios of 0.69 and 0.63, respectively) (153). In addition, some studies have reported a lower risk of cancer recurrence among antidepressant users (154), further emphasizing the complexity and heterogeneity of these associations.

These divergent findings likely stem from both methodological limitations and biological variability. A primary methodological issue is confounding by indication, wherein the apparent relationship between antidepressant use and poorer outcomes in certain cancers may reflect the underlying severity of depression rather than the pharmacologic effect of the drug itself. This factor is inherently difficult to control for in observational analyses. From a biological standpoint, specific antidepressants may exhibit direct antineoplastic activity. Preclinical evidence suggests that fluoxetine can radiosensitize glioma cells, inhibit STAT3-mediated metastasis in osteosarcoma, and reverse chemotherapy resistance (155–157).

In summary, the heterogeneous and sometimes paradoxical associations between antidepressant use and cancer outcomes highlight a substantial knowledge gap that warrants rigorous investigation. The current evidence base—subject to confounding and lacking causal inference—emphasizes the need for future studies employing robust analytic designs that minimize bias, such as propensity score matching, instrumental variable analysis, and randomized controlled trials. Until more definitive data become available, clinical practice should remain pragmatic and patient-centered. Treatment decisions must prioritize the effective management of depressive symptoms while maintaining vigilance for potential pharmacologic interactions with chemotherapeutic and targeted agents (158, 159). In the absence of conclusive evidence from randomized controlled trials (160, 161), a cautious yet proactive approach to antidepressant use remains an essential component of holistic cancer care.

#### 4.4.2 Collaborative care management for multimorbidity

Collaborative Care Management (CoCM) is a structured, team-based model that integrates mental health treatment into cancer care through coordinated, multidisciplinary collaboration. It brings together the expertise of a cancer specialist, a patient care coordinator, and a mental health professional to provide comprehensive and continuous support. The model operates through a triad of providers with distinct, complementary roles: the oncologist oversees medical management, the psychiatric consultant advises on diagnosis and complex treatment planning, and the embedded care manager functions as the operational core. The care process begins with systematic patient identification and assessment, followed by evidence-based psychosocial interventions. A key element of CoCM is the continuous monitoring of patient progress using standardized scales, with regular caseload reviews in which the entire team collaborates to refine treatment plans (162).

Despite its demonstrated efficacy, implementing CoCM in oncology settings presents substantial challenges that extend beyond traditional barriers such as funding limitations for non-billable services and workforce shortages (163). A deeper, systemic issue lies in the predominance of a cancer-centric paradigm within the oncologic care ecosystem, which inadvertently marginalizes mental health care. This focus often leads to poor adherence to CoCM principles and unclear role definitions among team members (164, 165). In clinical practice, this manifests as psychosocial screening schedules tied exclusively to cancer treatment milestones—rather than ongoing mental health needs—and the premature discontinuation of behavioral support once patients transition out of active oncologic therapy. The lack of standardized communication tools further undermines continuity of care and integration of psychosocial services. Furthermore, existing CoCM research has primarily been conducted in non-cancer populations, leaving important gaps in understanding how this model can be optimized for patients with cancer–depression multimorbidity. In particular, deeper insight into patient experiences, workflow design, and the use of digital health technologies is needed to facilitate collaboration and follow-up (162).

Sustainable implementation of CoCM in oncology depends on alignment with broader healthcare policy frameworks. The model embodies the core principles of value-based care by simultaneously improving patient outcomes and enhancing system efficiency. To close the persistent policy–practice gap, targeted policy-level reforms are essential. Chief among these is the development of alternative payment models that directly incentivize and reward integrated care delivery, thereby mitigating financial and structural barriers and fostering the widespread adoption of CoCM in oncology practice.

## 5 Summary and prospects

### 5.1 Limitations

This scoping review systematically maps and synthesizes the current evidence on the complex relationship between depression and cancer, with particular attention to epidemiological associations, shared pathophysiological mechanisms, and the implications of integrated management strategies.

However, several limitations warrant consideration. First, the included studies were geographically concentrated and limited to English-language publications, potentially excluding relevant data from underrepresented regions such as Southeast Asia and Africa. As a result, sociocultural and healthcare system factors unique to these regions—potentially influencing the interaction between depression and cancer prognosis—may not have been adequately captured. Second, as a scoping review, the included literature exhibited substantial heterogeneity in study design, sample composition, diagnostic criteria, depression measurement tools, and outcome reporting. This variability limited the ability to standardize data synthesis and may have influenced the overall interpretation of the association mechanisms. Third, most of the included studies were observational in nature, precluding causal inference. Although many attempted to adjust for major confounders, residual confounding remains a possibility. Finally, several studies lacked detailed information regarding depression severity, longitudinal symptom trajectories, and treatment status, constraining the ability to explore dose–response relationships or identify critical therapeutic windows.

### 5.2 Prospects

Addressing the evidence gaps and complexities identified in this review will require a coordinated, multidisciplinary approach that not only clarifies the bidirectional molecular underpinnings of the cancer–depression relationship but also translates this mechanistic insight into practical, patient-centered solutions. Future research must advance beyond observational associations to establish causal pathways through longitudinal cohort designs and innovative analytic approaches, such as Mendelian randomization, with the goal of identifying shared biological and therapeutic targets. Simultaneously, the field should prioritize the development and implementation of precision prevention strategies and multimodal interventions that integrate mental health management directly into oncologic care pathways. Such strategies should align with principles of personalized medicine, leveraging biological, behavioral, and psychosocial data to tailor prevention and treatment to individual patient profiles. Ultimately, the transformative potential of this work lies in the systematic validation of integrated care models that embed psychological health as an essential and inseparable component of high-quality cancer treatment. Achieving this integration will bridge the gap between mechanistic discovery and clinical practice, translating scientific understanding into measurable improvements in patient survival, functional recovery, and quality of life.

## Glossary

PHQ-8: Patient Health Questionnaire-8
HR: Hazard Ratio
ICD: International Classification of Diseases
PHQ-9: Patient Health Questionnaire-9
CI: Confidence Interval
DIS: Diagnostic Interview Schedule
RR: Relative Risk
SAS: Self-Rating Anxiety Scale
SDS: Self-Rating Depression Scale
RBC: red blood cell count
HADS: Hospital Anxiety and Depression Scale
RCDW: red cell distribution width
BDI-II: Beck Depression Inventory-II
MDD: major depressive disorder
BMI: body mass index
TCGA-BRCA: The Cancer Genome Atlas - Breast Invasive Carcinoma
ER: Estrogen Receptor
HPA axis: Hypothalamic-pituitary-adrenal axis
WGCNA: Weighted Gene Co-expression Network Analysis
TME: Tumor microenvironment
NE/E: Norepinephrine/Epinephrine
GC: Glucocorticoids
TLRs: Toll-like receptors
PAMPs: pathogen-associated molecular patterns
SAS: Sympathetic-adrenal-medullary system
PRRs: pattern recognition receptors
BDNF: brain-derived neurotrophic factor
IDO: Indoleamine 2,3-dioxygenase
5-HT: 5-Hydroxytryptamine (serotonin)
TME: Tumor microenvironment
DAMPs: damage-associated molecular patterns
MAPK: mitogen-activated protein kinase
NMDA: N-Methyl-D-aspartic acid
IL: Interleukin
TNF: Tumor Necrosis Factor
FGF2: fibroblast growth factor 2
VEGF: vascular endothelial growth factor
Gs-PKA: G protein (Gs) - Protein Kinase A (PKA)
PI3K-Akt: Phosphoinositide 3-Kinase (PI3K)-Akt
IFNγ: Interferon gamma
PD-1: Programmed cell death protein 1
BTLA: B- and T-lymphocyte Attenuator
DC: Dendritic Cell
COX-2/PGE2 pathway: Cyclooxygenase-2 (COX-2) - Prostaglandin E2 (PGE2) Pathway
NPY: neuropeptide Y
TAM: tumor-associated macrophage
IL-6/STAT3: Interleukin-6 (IL-6) - Signal Transducer and Activator of Transcription 3 (STAT3)
MDSCs: Myeloid-Derived Suppressor Cells
APC: Antigen-presenting cell
CRC: colorectal cancer
OR: Odds Ratio
NSCLC: non-small-cell lung cancer
MBE: Mind-Body Exercise
CTLA-4: Cytotoxic T Lymphocyte-Associated Antigen-4
DHIs: Digital Health Interventions
PD-L1: Programmed death-ligand 1
SSRIs: selective serotonin reuptake inhibitors
Tregs: Regulatory T Cells
RCTs: randomized controlled trials
PTSD: post-traumatic stress disorder
PUFAs: Omega-3 polyunsaturated fatty acids
CBT: Cognitive Behavioral Therapy
TREK-1: TWIK-Related K+ Channel 1
QoL: Quality of Life
TCR: T Cell Receptor
CoCM: Collaborative Care Management
AhR: Aryl Hydrocarbon Receptor
ILA: Indole-3-acetic acid
CTL: Cytotoxic T Lymphocyte
DC: Dendritic Cell
NK: Natural Killer cell
STING: Stimulator of Interferon Genes
IFN-γ: Interferon-gamma
MHC: Major Histocompatibility Complex
Th1: T helper cell type 1
FL: Fluoxetine.
