# Supplementary material for: New highlights in cancer and depression multimorbidity: a scoping systematic review
Source: Front Oncol. 2025 Dec 2;15:1674653. doi: 10.3389/fonc.2025.1674653 (PMC12705380; doi:10.3389/fonc.2025.1674653)
Supplement: Supplementary file 1 [file Table1.docx]

# Supplementary Material

**Table S1. PRISMA-ScR Checklist.**

| **SECTION** | **ITEM** | **PRISMA-ScR CHECKLIST ITEM** | **REPORTED IN SECTION #** |
| --- | --- | --- | --- |
| **TITLE** | | | |
| Title | 1 | Identify the report as a scoping review. | Title and 2.1 Study design |
| **ABSTRACT** | | | |
| Structured summary | 2 | Provide a structured summary that includes (as applicable): background, objectives, eligibility criteria, sources of evidence, charting methods, results, and conclusions that relate to the review questions and objectives. | Abstract section |
| **INTRODUCTION** | | | |
| Rationale | 3 | Describe the rationale for the review in the context of what is already known. Explain why the review questions/objectives lend themselves to a scoping review approach. | 1. Introduction |
| Objectives | 4 | Provide an explicit statement of the questions and objectives being addressed with reference to their key elements (e.g., population or participants, concepts, and context) or other relevant key elements used to conceptualize the review questions and/or objectives. | 1. Introduction |
| **METHODS** | | | |
| Protocol and registration | 5 | Indicate whether a review protocol exists; state if and where it can be accessed (e.g., a Web address); and if available, provide registration information, including the registration number. | N/A |
| Eligibility criteria | 6 | Specify characteristics of the sources of evidence used as eligibility criteria (e.g., years considered, language, and publication status), and provide a rationale. | 2.3 Eligibility Criteria |
| Information sources* | 7 | Describe all information sources in the search (e.g., databases with dates of coverage and contact with authors to identify additional sources), as well as the date the most recent search was executed. | 2.3 Eligibility Criteria |
| Search | 8 | Present the full electronic search strategy for at least 1 database, including any limits used, such that it could be repeated. | Supplementary material |
| Selection of sources of evidence† | 9 | State the process for selecting sources of evidence (i.e., screening and eligibility) included in the scoping review. | 2.5 Study Selection |
| Data charting process‡ | 10 | Describe the methods of charting data from the included sources of evidence (e.g., calibrated forms or forms that have been tested by the team before their use, and whether data charting was done independently or in duplicate) and any processes for obtaining and confirming data from investigators. | 2.6 Charting the data and quality assessment |
| Data items | 11 | List and define all variables for which data were sought and any assumptions and simplifications made. | N/A |
| Critical appraisal of individual sources of evidence§ | 12 | If done, provide a rationale for conducting a critical appraisal of included sources of evidence; describe the methods used and how this information was used in any data synthesis (if appropriate). | 2.6 Charting the data and quality assessment |
| Synthesis of results | 13 | Describe the methods of handling and summarizing the data that were charted. | 2.6 Charting the data and quality assessment |
| **RESULTS** | | | |
| Selection of sources of evidence | 14 | Give numbers of sources of evidence screened, assessed for eligibility, and included in the review, with reasons for exclusions at each stage, ideally using a flow diagram. | 3. Results and Figure 1 |
| Characteristics of sources of evidence | 15 | For each source of evidence, present characteristics for which data were charted and provide the citations. | 3.1 Study characteristics |
| Critical appraisal within sources of evidence | 16 | If done, present data on critical appraisal of included sources of evidence (see item 12). | Supplementary material TableS3 |
| Results of individual sources of evidence | 17 | For each included source of evidence, present the relevant data that were charted that relate to the review questions and objectives. | Supplementary material TableS4 |
| Synthesis of results | 18 | Summarize and/or present the charting results as they relate to the review questions and objectives. | 3.2 Increased Mortality Risk and Table1 |
| **DISCUSSION** | | | |
| Summary of evidence | 19 | Summarize the main results (including an overview of concepts, themes, and types of evidence available), link to the review questions and objectives, and consider the relevance to key groups. | Section 4.1 to 4.4 |
| Limitations | 20 | Discuss the limitations of the scoping review process. | 5.1 Limitations |
| Conclusions | 21 | Provide a general interpretation of the results with respect to the review questions and objectives, as well as potential implications and/or next steps. | 5.2 Prospects |
| **FUNDING** | | | |
| Funding | 22 | Describe sources of funding for the included sources of evidence, as well as sources of funding for the scoping review. Describe the role of the funders of the scoping review. | Funding section |

JBI = Joanna Briggs Institute; PRISMA-ScR = Preferred Reporting Items for Systematic reviews and Meta-Analyses extension for Scoping Reviews.

* Where *sources of evidence* (see second footnote) are compiled from, such as bibliographic databases, social media platforms, and Web sites.

† A more inclusive/heterogeneous term used to account for the different types of evidence or data sources (e.g., quantitative and/or qualitative research, expert opinion, and policy documents) that may be eligible in a scoping review as opposed to only studies. This is not to be confused with *information sources* (see first footnote).

‡ The frameworks by Arksey and O’Malley (6) and Levac and colleagues (7) and the JBI guidance (4, 5) refer to the process of data extraction in a scoping review as data charting*.*

§ The process of systematically examining research evidence to assess its validity, results, and relevance before using it to inform a decision. This term is used for items 12 and 19 instead of "risk of bias" (which is more applicable to systematic reviews of interventions) to include and acknowledge the various sources of evidence that may be used in a scoping review (e.g., quantitative and/or qualitative research, expert opinion, and policy document).

*From:* Tricco AC, Lillie E, Zarin W, O'Brien KK, Colquhoun H, Levac D, et al. PRISMA Extension for Scoping Reviews (PRISMAScR): Checklist and Explanation. Ann Intern Med. 2018;169:467–473. [doi: 10.7326/M18-0850](http://annals.org/aim/fullarticle/2700389/prisma-extension-scoping-reviews-prisma-scr-checklist-explanation).

**Table S2. Search strategy.**

**Web of science:**

cancer OR neoplasm* OR tumor* OR carcinoma

AND

depression OR depressive OR "depressive disorder" OR "mood disorder*"

AND

prevalence OR incidence OR "risk factor*" OR mortality OR "clinical outcome*" OR "treatment outcome*"

**Pubmed:**

( (cancer[Title/Abstract] OR neoplasm*[Title/Abstract] OR tumor*[Title/Abstract] OR carcinoma*[Title/Abstract]) AND (depression[Title/Abstract] OR depressive[Title/Abstract] OR "depressive disorder"[Title/Abstract]) AND (prevalence[Title/Abstract] OR incidence[Title/Abstract] OR "risk factor*"[Title/Abstract] OR mortality[Title/Abstract] OR "clinical outcome*"[Title/Abstract] OR "treatment outcome*"[Title/Abstract]) ) AND 2020:2025[pdat] AND english[la] AND "journal article"[pt]

**Scopus:**

cancer OR neoplasm* OR tumor* OR carcinoma

AND

depression OR depressive OR "depressive disorder" OR "mood disorder*"

AND

prevalence OR incidence OR "risk factor*" OR mortality OR "clinical outcome*" OR "treatment outcome*"

**Embase:**

cancer OR neoplasm* OR tumor* OR carcinoma

AND

depression OR depressive OR "depressive disorder" OR "mood disorder*"

AND

prevalence OR incidence OR "risk factor*" OR mortality OR "clinical outcome*" OR "treatment outcome*"

**Table S3. Quality assessment of included reports*.**

| Authors | Selection | Comparability | Exposure | Total |
| --- | --- | --- | --- | --- |
| Yang G et al. | **4** | **1** | **2** | **7** |
| He J, Zhang Y | **4** | **2** | **3** | **9** |
| Trudel-Fitzgerald C et al. | **4** | **2** | **3** | **9** |
| Orive M et al. | **4** | **2** | **2** | **8** |
| Shim EJ et al. | **4** | **1** | **3** | **8** |
| Vilalta-Lacarra A et al. | **4** | **1** | **3** | **8** |
| Paredes AZ et al. | **4** | **1** | **3** | **8** |
| Chierzi Fet al. | **4** | **1** | **3** | **8** |
| Aboumrad M et al. | **4** | **2** | **3** | **9** |
| Oh TK et al. | **4** | **2** | **3** | **9** |
| Bach L et al. | **4** | **1** | **1** | **6** |
| Miller NE et al. | **4** | **1** | **3** | **8** |
| Walker J et al. | **4** | **1** | **3** | **8** |
| Sathianathen NJ et al. | **3** | **1** | **3** | **7** |
| Rumalla K et al. | **3** | **1** | **3** | **7** |
| Tao F et al. | **4** | **2** | **2** | **8** |
| Adeyemi OJ et al. | **4** | **1** | **3** | **8** |
| Ouh YT et al. | **4** | **1** | **3** | **8** |
| Tan PX et al. | **4** | **1** | **3** | **8** |
| Qian Z et al. | **4** | **1** | **3** | **8** |
| Lei F et al. | **4** | **2** | **3** | **9** |
| Varela-Moreno E et al. | **4** | **2** | **3** | **9** |
| Sundar S et al. | **4** | **1** | **1** | **6** |
| Walker J et al. | **4** | **1** | **3** | **8** |
| Kuczmarski TM et al. | **4** | **1** | **3** | **8** |
| Crump C et al. | **4** | **1** | **3** | **8** |
| Herweijer E et al. | **4** | **2** | **3** | **9** |
| Leung B et al. | **4** | **1** | **2** | **7** |
| Hu S et al. | **4** | **1** | **2** | **7** |
| Li YZ et al. | **4** | **2** | **3** | **9** |
| Davis NE et al. | **4** | **1** | **3** | **8** |
| McFarland DC et al. | **4** | **1** | **3** | **8** |
| Sanghvi DE et al. | **4** | **1** | **1** | **6** |
| Gallagher TJ et al. | **4** | **2** | **1** | **7** |
| Sancassiani Fet al. | **4** | **1** | **1** | **6** |
| Zhou L et al. | **4** | **2** | **3** | **9** |

**Table S4. Characteristics of included reports (n=36).**

| Authors | Year | Country | Study Design | Study Population | Exposure Factors | Outcome Measures | Key Findings / Effect Measures |
| --- | --- | --- | --- | --- | --- | --- | --- |
| Yang G et al. | 2022 | China | Retrospective cohort study | 234 Pituitary adenoma (PA) patients undergoing surgery | SDS > 52 defined as depression | Cure/effective/recurrence | Perioperative depressive symptoms (SDS >52) was an independent risk factor for poor prognosis in pituitary adenoma patients (Adjusted OR = 2.504, 95% CI: 1.418–7.458, *p*=0.003). |
| He J, Zhang Y | 2023 | China | Retrospective cohort study | 258 ovarian cancer patients undergoing surgery | SDS > 52 or SAS > 50 defined as negative emotion | 2-year/3-year survival rate, recurrence rate | Perioperative negative emotions were independently associated with poorer survival, with lower 2-year (65.1% vs 80.9%) and 3-year (44.2% vs 65.1%) survival rates (Adjusted OR = 0.256, 95% CI: 0.098–0.672, *p* =0.006). |
| Trudel-Fitzgerald C et al. | 2020 | USA | Prospective cohort study | 1,732 colorectal cancer patients | Comprehensive anxiety/depression symptoms, diagnosis, medication use | All-cause mortality, CRC-specific mortality | Both anxiety and depression symptoms were associated with increased all-cause mortality (HR per 1-SD increase: 1.16 for each). Clinical depression was significantly linked to higher mortality (HR = 1.28, 95% CI: 1.06–1.56). |
| Orive M et al. | 2022 | Spain | Prospective observational cohort study | 2,531 colorectal cancer patients | HADS-A/HADS-D, EQ-5D-5L, EORTC QLQ-C30 | 5-year all-cause mortality | Higher HADS-Anxiety (OR=1.072 (1.046 – 1.099), *p* <0.0001) and HADS-Depression (OR=1.118 (1.092 – 1.145), *p* <0.0001) scores were independently associated with increased one-year mortality risk . Worse HRQoL scores also predicted mortality. |
| Shim EJ et al. | 2020 | South Korea | Retrospective cohort study | 124,381 breast cancer patients | ICD-10 diagnosis of depression/anxiety, antidepressant treatment | All-cause mortality | Depression (HR=1.26, 95%CI 1.18-1.36), anxiety (HR=1.14, 95% CI 1.08-1.22), and their comorbidity (HR=1.38, 95% CI 1.24-1.54) were associated with increased all-cause mortality. Antidepressant treatment was associated with a reduction in this excess risk. |
| Vilalta-Lacarra A et al. | 2023 | Spain | Community-based cohort study | 5,646 community adults | PHQ-9 assessed depression phenotypes, antidepressant use | Cancer mortality, non-cancer mortality | Cognitive/affective depressive symptoms were associated with cancer mortality in both men (HR=2.23, 95% CI = 1.11-4.44) and women (HR=3.69, 95% CI = 1.69-8.09). SSRI use was associated with both cancer and non-cancer mortality in men. |
| Paredes AZ et al. | 2021 | USA | Population-based retrospective cohort study | 54,234 pancreatic cancer patients | ICD diagnosis of mental illness (depression, anxiety, etc.) | All-cause mortality, pancreatic cancer-specific mortality | Patients with pre-existing mental illness, particularly severe disorders (bipolar/schizophrenia), had lower rates of curative surgery and higher all-cause (HR=1.20, 95% CI 1.21-1.40) and pancreatic cancer-specific (HR=1.27, 95% CI 1.17-1.37) mortality. |
| Chierzi Fet al. | 2023 | Italy | Retrospective cohort study | 101,487 common mental disorder patients | ICD-9-CM diagnosis of depression/anxiety etc. | All-cause mortality, cancer mortality | Patients with Common Mental Disorders had significantly elevated mortality from all cancers combined (SMR=2.08, 95% CI 2.01-2.16) and from several specific cancer sites compared to the general population. |
| Aboumrad M et al. | 2025 | USA | National retrospective cohort study | 6,051 female veterans with breast cancer | ICD diagnosis of major depressive disorder | Breast cancer recurrence, breast cancer-specific mortality | MDD was associated with an increased risk of breast cancer recurrence (HR=1.37, 95% CI = 1.19 to 1.57) and breast cancer-specific mortality (HR=1.30, 95% CI = 1.02 to 1.64). This risk was heightened in smokers and those with substance use disorder. |
| Oh TK et al. | 2021 | South Korea | National retrospective cohort study | 4,275 brain tumor resection patients | ICD-10 diagnosis of postoperative depression | 2-year all-cause mortality, brain cancer mortality | Post-craniotomy depression was associated with increased 2-year all-cause (HR=1.58, 95% confidence interval: 1.38-1.80; *p* < 0.001) and brain cancer-specific (HR=1.68, 95% CI：1.39–2.04; *p* < 0.001) mortality, indicating its role as a significant postoperative prognostic factor. |
| Bach L et al. | 2021 | UK | Retrospective cohort study | 6,656 breast cancer women | ICD-10 diagnosis of depression, anxiety, sleep disorders | 5-year all-cause mortality | Both depression (HR=1.44, 95% CI: 1.17-1.78) and sleep disorders (HR=1.37, 95% CI: 1.02-1.84) were independently associated with increased 5-year all-cause mortality. |
| Miller NE et al. | 2024 | UK | Population-based prospective cohort study | 1,352 older cancer survivors | CES-D defined as high depressive symptoms | All-cause mortality, cancer-specific mortality | High depressive symptoms were associated with increased all-cause mortality within 8 years of diagnosis, most strongly in the early years (<4 years: HR=1.93, 95%CI = 1.52-2.45). Socioeconomic position did not moderate this association. |
| Walker J et al. | 2020 | UK | Multicenter retrospective cohort study | 19,966 patients with five common cancers | HADS assessed depression and anxiety symptoms | All-cause mortality | Depressive symptoms consistently predicted poorer survival. After adjusting for depression, anxiety was not associated with survival in men and was associated with better survival in women, indicating distinct prognostic roles. |
| Sathianathen NJ et al. | 2019 | USA | Population-based retrospective cohort study | 66,476 localized bladder cancer patients | ICD-9-CM diagnosis of mental illness | Overall survival, disease-specific survival | Patients with severe mental illness (OR=0.55, 95% CI 0.37-0.81) or depression (OR=0.71, 95% CI 0.58-0.88) were significantly less likely to receive curative-intent therapy. Among these patients, those who underwent radical cystectomy achieved significantly better overall (HR=0.54, 95% CI 0.43-0.67) and disease-specific survival (HR=0.76, 95% CI 0.58-0.99). |
| Rumalla K et al. | 2020 | USA | National retrospective cohort study | 57,621 malignant brain tumor surgeries | ICD-9 diagnosis of major depressive disorder | Complications, readmission rates | The presence of MDD was associated with nonroutine discharge (odds ratio, 1.10-125; *p* < 0.0001) as well as higher rates of neurologic complications (odds ratio, 1.03-1.18; *p* = 0.003). |
| Tao F et al. | 2023 | China | Prospective cohort study | 178 advanced gastric cancer patients receiving chemotherapy | SAS/SDS > 50 defined as negative emotions | PFS, OS, quality of life | Negative emotions were prevalent and associated with shorter PFS and OS. They were an independent risk factor for OS (HR=0.702, 95%CI=0.497- 0.997, *p* =0.045) and were linked to worse quality of life. |
| Adeyemi OJ et al. | 2021 | USA | Retrospective cohort study | 2,819 breast cancer women | Kessler-6 defined as psychological distress | Overall survival time | Self-reported psychological distress (aHR=1.46, 95% CI：1.02-2.09) and poorer self-rated health (aHR=3.50, 95% CI：2.61-4.69) were significantly associated with shorter survival times in women with breast cancer. |
| Ouh YT et al. | 2025 | South Korea | National retrospective cohort study | 85,327 gynecologic cancer patients | ICD-10 diagnosis of depression/anxiety | All-cause mortality | Depression alone (OR=1.46, 95% CI 1.27-1.66) and comorbid depression/anxiety (OR=1.47, 95% CI 1.31-1.65) were independent predictors of higher all-cause mortality. Anxiety alone was not significantly associated with mortality. |
| Tan PX et al. | 2025 | China | Single-center retrospective cohort study | 319 esophageal cancer MIE patients | PHQ-9 assessed postoperative depression | Recurrence-free survival | ASSO and Cox regression identified clinical stage (HR=2.472, *p* =0.003), the preoperative systemic inflammatory index (SII, HR=1.001, P<0.001), and depressive symptoms severity (HR=2.398, *p* =0.004) as independent predictors of RFS. |
| Qian Z et al. | 2021 | China | Single-center prospective cohort study | 103 NMIBC patients | HADS-D defined as depressive symptoms | 1-year recurrence rate | Depressive symptoms (HR=2.493, 95%CI：1.048-5.930，*p* =0.039), but not anxiety, were an independent risk factor for 1-year recurrence in NMIBC, with the strongest effect observed in intermediate-risk patients. |
| Lei F et al. | 2023 | USA | Population-based retrospective cohort study | 6,054 breast cancer patients | ICD-9-CM diagnosis of depression (pre/post diagnosis) | Overall survival, treatment receipt | Post-diagnosis depression was associated with worse overall survival (HR=1.51, 95% CI, 1.24-1.83). A trend suggested lower odds of receiving guideline-concordant treatment among those with pre-diagnosis depression. |
| Varela-Moreno E et al. | 2022 | Spain | Multicenter prospective observational cohort study | 2,602 colorectal cancer patients | HADS-D >8 defined as depressive symptoms | 5-year overall survival | Depressive symptoms at diagnosis were an independent predictor of worse 5-year overall survival (HR=1.47, 95% CI：1.21-1.8, *p* =0.002) in a large, prospective multicenter cohort of colorectal cancer patients. |
| Sundar S et al. | 2023 | UK | Single-center prospective cohort study | ~300 prostate cancer patients | HADS assessed psychological distress | All-cause mortality | Baseline anxiety (HR=2.38, 95%CI=1.28 - 4.44) and post-treatment depressive symptoms (HR=3.18, 95%CI=1.37 - 7.35) were significantly associated with increased long-term (11-year) all-cause mortality in prostate cancer patients. |
| Walker J et al. | 2021 | UK | Multicenter prospective cohort study | 20,582 patients with five cancers | SCID diagnosis of major depression | All-cause mortality, cancer-specific mortality | Major Depression, ascertained via structured clinical interview, was consistently associated with increased all-cause mortality across five common cancer types (Pooled HR=1.41, 95% CI: 1.29–1.54). |
| Kuczmarski TM et al. | 2023 | USA | Population-based retrospective cohort study | 13,244 DLBCL patients | ICD-9-CM diagnosis of depression/anxiety | 5-year overall survival, lymphoma-specific survival | Pre-existing depression was a significant independent predictor of markedly inferior survival in patients with diffuse large B-cell lymphoma (DLBCL). Those with depression alone faced the greatest risk, with a 37% increase in all-cause mortality (HR 1.37, 95% CI 1.28-1.47) and a similarly elevated 37% increase in lymphoma-specific mortality (HR 1.37, 95% CI 1.26-1.49) compared to patients with no mental health disorder. |
| Crump C et al. | 2024 | Sweden | National cohort study | 180,189 prostate cancer patients | ICD-10 diagnosis of major depression | All-cause mortality, prostate cancer-specific mortality | After adjusting for sociodemographic factors and comorbidities, major depression was associated with significantly higher all-cause mortality in men with high-risk PC (HR, 1.50; 95% CI, 1.44-1.55) or low- or intermediate-risk PC (1.64; 1.56-1.71). |
| Herweijer E et al. | 2023 | Sweden | National registry-based cohort study | 20,177 cervical cancer patients | ICD diagnosis of mental disorders | All-cause mortality, cervical cancer-specific mortality | Pre-existing mental disorders were associated with worse overall survival (fully adjusted HR=1.19, 95% CI，1.06-1.34). The association with cervical cancer-specific mortality was attenuated after full adjustment for cancer stage and sociodemographics. |
| Leung B et al. | 2021 | Canada | Retrospective cohort study | 25,382 geriatric cancer patients | PSSCAN-R assessed anxiety/depression/social isolation | Overall survival | Anxiety (HR=1.30, 95%CI=1.24–1.37), depressive symptoms (HR=1.51, 95%CI=1.43–1.59), and social isolation (HR=1.12, 95%CI=1.07–1.67) were independent predictors of shorter overall survival in geriatric cancer patients. |
| Hu S et al. | 2021 | China | Prospective cohort study | 194 prostate cancer post-surgery patients | SAS/SDS defined as anxiety/depression | Disease-free survival, overall survival | Post-prostatectomy anxiety and depression symptoms worsened over time. Baseline depression was an independent predictor of worse disease-free survival (HR=1.796, 95%CI=1.069–3.018, *p* =0.027). |
| Li YZ et al. | 2022 | China | Prospective cohort study | 598 ovarian cancer patients | PHQ-9 defined as depression, GAD-7 as anxiety | All-cause mortality | Prediagnosis depressive symptoms (HR=2.10, 95% CI：1.20−3.70), but not anxiety symptoms, were independently associated with increased all-cause mortality. Comorbid symptoms showed the highest risk. |
| Davis NE et al. | 2022 | USA | Retrospective case-control study | 856 pancreatic cancer patients | ICD/symptoms/medication defined prediagnosis depression/anxiety | Overall survival, treatment compliance | Prodromal depression/anxiety were associated with a lower likelihood of receiving chemotherapy (OR=0.58, *p* = 0.04) and, in metastatic patients, with shorter overall survival (HR=1.32, *p* = 0.04). |
| McFarland DC et al. | 2021 | USA | Retrospective case cohort study | 123 metastatic lung cancer patients | HADS-D defined as depression, CRP mg/dl as inflammation | Overall survival | Both depressive symptoms (HR=1.12, 95% CI：1.05-1.179) and inflammation (CRP≥1; HR=2.85, 95% CI：1.856-4.388) were independent risk factors for shorter survival. Depression partially mediated the effect of inflammation on survival. |
| Sanghvi DE et al. | 2024 | USA | Prospective cohort study | 2,342 cancer patients | CES-D assessed depression trajectories (four types) | Mortality | Longitudinal depression trajectories were strong predictors of mortality. The incident (worsening) trajectory carried the highest risk (OR=6.89, 95% CI = 4.55-10.43), followed by recovering and chronic trajectories. |
| Gallagher TJ et al. | 2025 | USA | Retrospective cohort study | 258,259 head and neck cancer survivors | ICD diagnosis of anxiety/depression, treatment modalities | Mortality | Treatment for anxiety/depression, particularly psychotherapy (HR=0.75, 95% CI：0.68-0.82), was associated with a significant reduction in all-cause mortality among Head and Neck Cancer survivors. |
| Sancassiani Fet al. | 2021 | Italy | Longitudinal cohort study | 263 cancer patients | PHQ-9 defined as MDD | Early death within 9 months | MDD was associated with a significantly increased risk of premature death within 9 months (RR=2.15, 95% CI: 1.10–4.20) in a mixed cancer cohort. |
| Zhou L et al. | 2021 | China | Longitudinal cohort study | 302 colorectal cancer patients | HADS-A/HADS-D defined as anxiety/depression | Overall survival | Anxiety and depressive symptoms were persistent post-surgery. Depression at multiple timepoints, particularly at 2 years (HR=1.99, *p* =0.003), was an independent prognostic factor for worse overall survival. |
